# Die Novelle der Ärzteausbildungsordnung zur Weiterbehandlung durch Kinder- und Jugendpsychiater:innen über die Volljährigkeit hinaus: Eine qualitative Analyse von Einstellungen und klinischer Realität

**DOI:** 10.1007/s40211-025-00527-5

**Published:** 2025-05-30

**Authors:** Matthias Neumann, Sylvia Dörfler, Anita Holzinger, Verena Steiner-Hofbauer

**Affiliations:** 1 Research Centre Transitional Psychiatry, Karl Landsteiner University of Health Sciences, Dr. Karl-Dorrek-Straße 30, 3500 Krems, Österreich; 2 Research Unit for Curriculum Development, Medical University of Vienna, Waehringer Gürtel 18-20, 1090 Wien, Österreich; 3 Doctoral Programme Meduni Vienna, Medical University of Vienna, Spitalgasse 23, 1090 Wien, Österreich

**Keywords:** Kinder- und Jugendpsychiatrie, Transitionsalter, Versorgung, Ärztinnen-/Ärzte-Ausbildungsordnung, Österreich, Children and adolescent psychiatry, Transition age, Care, Amendment, Austria

## Abstract

**Hintergrund:**

Psychisch erkrankte Jugendliche benötigen im Übergang zum Erwachsenenalter stabile Versorgungsstrukturen. Der traditionell vorgeschriebene Behandlungswechsel mit 18 Jahren von der kinder- und jugendpsychiatrischen in die erwachsenenpsychiatrische Versorgung wurde diesem Bedarf nicht gerecht. Eine am 15. Mai 2024 in Österreich in Kraft getretene Novelle der Ärztinnen-/Ärzte-Ausbildungsordnung ermöglicht Kinder- und Jugendpsychiater:innen (KJPs) in Österreich offiziell, dass sie ihre Patient:innen über die Volljährigkeitsgrenze hinaus weiterbehandeln dürfen. Die vorliegende Studie untersucht erstmalig die Einschätzungen von KJPs zur Novelle und erhebt erste praktische Auswirkungen in der Versorgung.

**Methodologie:**

Es wurden semi-strukturierte Interviews mit 16 KJPs aus verschiedenen Arbeitskontexten durchgeführt. Die Datenauswertung erfolgte mittels Reflexiver Thematischer Analyse.

**Ergebnisse:**

Die Studie zeigt, dass die Novelle für viele KJPs überraschend eingeführt wurde, wobei vor allem die fehlende Vorlaufzeit bei einigen niedergelassenen KJPs für Unmut sorgte. Inhaltlich wurde sie jedoch überwiegend positiv bewertet, von einigen sogar als längst überfällig angesehen. Viele KJPs sehen sich aufgrund der hohen Auslastung weiterhin gezwungen, ihre begrenzten Ressourcen vorrangig für Minderjährige einzusetzen, woran auch die Novelle nichts ändere. Aufgrund des hohen Bettendrucks in Kliniken habe die Novelle auch im stationären Bereich noch nicht zu nennenswerten Veränderungen geführt.

**Fazit für die Praxis:**

Die Novelle findet bei den befragten KJPs inhaltlich Zustimmung, jedoch verhindert der vorhandene Ressourcenmangel sowohl im stationären als auch im ambulanten Bereich die Realisierung des Potenzials. Ohne Kapazitätsausbau werden die durch die Novelle intendierten Verbesserungen der psychiatrischen Versorgung für Patient:innen im Transitionsalter voraussichtlich weitgehend ausbleiben.

## Abstract (en)

**Background:**

Adolescents with mental health conditions need stable care structures during their transition to adulthood. The traditionally mandated transfer from child and adolescent psychiatry to adult psychiatric care at the age of 18 does not satisfy this need. A new amendment to the medical training regulations (Ärztinnen/Ärzte-Ausbildungsordnung), effective May 15, 2024, now officially allows child and adolescent psychiatrists (CAPs) in Austria to continue treatment after their patients reach legal adulthood. This study is the first to analyze CAPs’ assessments of the amendment and aims to document initial effects on care provision.

**Methodology:**

Semi-structured interviews were conducted with 16 child and adolescent psychiatrists (CAPs) working in various settings. Data analysis was performed using Reflexive Thematic Analysis.

**Results:**

The study shows that the amendment was introduced unexpectedly for many CAPs, with some private practitioners expressing frustration over the lack of preparation time. Despite this, the reform was largely welcomed and even considered overdue by some. However, given the high patient load, many CAPs still allocate their limited resources to minors, leaving the amendment’s impact minimal. In the inpatient sector, persistent shortages of beds have also hindered any substantial changes.

**Conclusions:**

While the amendment receives substantive approval from the interviewed CAPs, existing resource constraints in both inpatient and outpatient sectors prevent the realization of its potential. The improvements in psychiatric care for transition-age patients intended by the amendment will likely not materialize without capacity expansion.

## Hintergrund

Der Übergang von der Adoleszenz ins Erwachsenenalter ist mit einer Reihe wichtiger Entwicklungsaufgaben verbunden [1, 2], deren Bewältigung für Jugendliche mit psychischen Erkrankungen eine besondere Herausforderung darstellt. Zur effektiven Bewältigung dieser sind eine stabile Umgebung und eine angemessene soziale Unterstützung von großer Bedeutung [3, 4]. Das bestehende österreichische psychiatrische Versorgungssystem trägt diesem jedoch oft unzureichend Rechnung, da die Versorgungskontinuität im Regelfall durch einen abrupten Wechsel von der kinder- und jugendpsychiatrischen (KJP) zur erwachsenenpsychiatrischen (EP) Behandlung mit dem 18. Geburtstag unterbrochen wird. Fehlende Kontinuität in der psychiatrischen Versorgung wurde bereits vielfach kritisiert [5–8], da sie häufig Behandlungslücken zur Folge hat [9–11]. Neben der Veränderung der rechtlichen Rahmenbedingungen der Behandlung durch das Erreichen der Volljährigkeit stellt auch der Wechsel von einer entwicklungs- und familienorientierten Kultur mit stark sozialpädagogischer Komponente zu einem in der EP meist sehr auf das Individuum fokussierten Behandlungsansatz eine besondere Schwierigkeit dar [11]. Die umfangreichste Studie zur Evaluation des Gelingens der Transition in Österreich stammt von Pollak et al. [12], deren Ergebnisse zeigen, dass nahezu alle befragten Kliniker:innen (98,8 %, *n* = 98) das vorhandene Transitionssystem als problematisch einstufen, wobei besonders Schnittstellenverluste bei der Weitergabe individueller Behandlungsgeschichten (63,7 % der EP-Kliniker:innen) beklagt werden. Für den Kinder- und Jugendbereich stellen vor allem das Finden geeigneter Weiterbehandlungsmöglichkeiten (85,3 %) sowie der abrupte Wegfall altersspezifischer Betreuungs- und Unterstützungsangebote die größten Herausforderungen dar [12].

Mit dem Ziel einer Verbesserung der Gestaltung dieser Übergangsphase kam es am 15. Mai 2024 zu einer Novelle der österreichischen Ärztinnen-/Ärzte-Ausbildungsordnung (ÄAO), die auf der Website des Bundesministeriums für Soziales Gesundheit, Pflege und Konsumentenschutz (BMSGPG) mit der Überschrift „Jugendpsychiatrie darf Patient:innen künftig bis zum Alter von 25 behandeln“ verkündet wurde [13]. In der ÄAO 2025 Anl. 14 A – Definition des Aufgabengebiets heißt es nun im Wortlaut [neuer Teil in kursiv]:

Das Sonderfach Kinder- und Jugendpsychiatrie und Psychotherapeutische Medizin umfasst die Prävention, Diagnostik, Behandlung einschließlich Psychotherapeutischer Medizin und Rehabilitation von im Kindes- und Jugendalter auftretenden psychischen Krankheiten, Störungen und Verhaltensauffälligkeiten einschließlich der psychiatrischen Behandlung von entwicklungsbedingten psychischen Erkrankungen sowie die fachspezifische Begutachtung, *wie auch erforderlichenfalls bei spezifischen Krankheitsbildern eine Weiterversorgung von bereits bestehenden Erkrankungen der in Behandlung stehenden Personen im Erwachsenenalter bis zur möglichen adäquaten Behandlungsübernahme durch Fachärztinnen/Fachärzte für Psychiatrie und Psychotherapeutische Medizin* [14].

Hierbei ist anzumerken, dass die mediale Verkündung der Novelle eine altersbezogene Grenze von 25 Jahren explizierte, wohingegen in der tatsächlichen Neufassung der ÄAO keine konkrete Altersangabe gemacht wurde. Hervorzuheben ist außerdem, dass die Novelle bereits am Tag der Verkündigung in Kraft getreten ist.

### Die vorliegende Studie

Die nun in der ÄAO verankerte Erweiterung des Aufgabengebiets für Kinder- und Jugendpsychiater:innen (KJPs) führt zu einer Ausweitung des Behandlungsalters, welche sich auch entsprechend in der Behandlungspraxis niederschlagen kann. Die vorliegende Studie ist die erste, welche die Einstellungen der KJPs zu dieser Novelle untersucht und erste Einblicke in mögliche praktische Auswirkungen dieser liefern soll.

## Methodologie

### Studiendesign

In der Studie kam ein qualitatives Design mit halbstrukturierten Interviews zum Einsatz. Die Auswertung der Daten erfolgte mittels Reflexiver Thematischer Analyse (RTA) nach Braun und Clarke [15, 16]. Entsprechend wird die Studie nach den von ihnen herausgegebenen Richtlinien zur reflexiven Analyseberichterstattung [17] präsentiert.

### Teilnehmende

Alle Studienteilnehmer:innen mussten zum Befragungszeitpunkt approbierte und in Österreich praktizierende Fachärzt:innen der KJP sein. Zusätzlich mussten sie zum Erhebungszeitpunkt bereits mindestens zwei Jahre in Österreich praktizieren, um potenzielle Auswirkungen der Novelle fundiert einschätzen zu können. KJPs in Ausbildung wurden von der Teilnahme ausgeschlossen, wohingegen Arbeit in leitender Funktion kein Ausschlusskriterium war. Es konnten insgesamt 16 KJPs (9 männlich, 7 weiblich) aus allen Bundesländern mit Ausnahme des Burgenlandes gewonnen werden. Auf eine detaillierte Beschreibung der Teilnehmer:innen wird verzichtet, da in Anbetracht der überschaubaren Anzahl an KJPs bereits die Kombination weniger demographischer oder berufsbezogener Merkmale Rückschlüsse auf einzelne Personen ermöglichen könnte – ein Aspekt, der von mehreren Befragten noch vor Beginn der Aufnahme mit Nachdruck hervorgehoben wurde.

### Rekrutierung

Der Erstautor (MN) kontaktierte insgesamt 88 potenziell geeignete KJPs via E‑Mail. Von diesen antworteten 68 nicht, vier lehnten ab und 16 erklärten sich bereit, an der Studie teilzunehmen. Die Teilnahme an der Studie wurde mit einer Aufwandsentschädigung von 70 € honoriert, wobei drei Teilnehmer:innen freiwillig auf diese verzichteten.

### Vorgehensweise

Allen Teilnehmenden wurde im Rahmen einer informierten Einwilligung vor Beginn des Interviews mitgeteilt, dass die durchgeführten Interviews aufgezeichnet werden und sie jederzeit ohne Angabe von Gründen ihre Studienteilnahme abbrechen können. Vor der Durchführung der Interviews wurde ein Leitfaden erstellt, der jedoch in Abhängigkeit der von den KJPs aufgebrachten Themen sowie deren Arbeitsbereichen nur sehr lose befolgt und auch laufend angepasst wurde.

Die Interviews wurden von MN, der keinen vorherigen Kontakt zu den Teilnehmenden hatte, telefonisch durchgeführt. Der Erhebungszeitraum erstreckte sich von 18.10.2024 bis 23.12.2024. Sämtliche Interviews, mit einer Dauer von 17 bis 25 min (*M* = 21 min), wurden aufgezeichnet, transkribiert und pseudonymisiert.

### Datenauswertung

Die Datenauswertung erfolgte nach den Prinzipien der RTA [15, 16]. Auf epistemologischer Ebene liegt dem Verfahren ein konstruktivistischer Ansatz zugrunde. Bedeutung und Erfahrung wurden dementsprechend als sozial konstruiert und in der Interaktion subjektiver und intersubjektiver Konstruktionsprozesse fortlaufend reproduziert verstanden. Die Datenanalyse erfolgte primär induktiv, das heißt, die Daten wurden offen kodiert und der Fokus lag auf teilnehmer- und datenbasierten Bedeutungen anstelle von vorab definierten Kategorien. Ergänzend kam eine deduktive Analyse zum Einsatz, um die Relevanz der durch offene Kodierung gewonnenen Themen sicherzustellen. In der Analyse wurde sowohl semantische als auch latente Kodierung eingesetzt. Entsprechend dem interpretativen Charakter der RTA wurde die Analyse von MN durchgeführt, eine auf eine vollständige Codierung durch mehrere Autoren samt Angabe von Interrater-Reliabilitäten wurde bewusst verzichtet, da das RTA-Framework auf einem konstruktivistischen Ansatz basiert und kein positivistischer Ansatz suggeriert werden soll. Die Analyseergebnisse spiegeln somit die Dateninterpretation von MN wider. Die Zweitautorin (SD) wurde im Codierungs- und Interpretationsprozess hinzugezogen, um den reflexiven Analyseprozess zu prüfen, Ideen auf Plausibilität zu testen und kollaborativ verschiedene Interpretationen der Daten zu erkunden. Die Codierung wurde mit MAXQDA Version 24.7.0 durchgeführt.

## Ergebnisse

### Thema 1: Und plötzlich war sie da – die Novelle ohne Vorlaufzeit

Noch bevor Einstellungen zur Novelle und mögliche Folgen dieser diskutiert werden konnten, brachten mehrere Teilnehmende großen Unmut über die Kommunikationsstrategie der Regierung zum Ausdruck. Der Großteil der Befragten habe von der Novelle über die Medien erfahren und sei von dieser überrascht gewesen.

Ich habe es erfahren über den ORF und ich weiß es nicht, ob es im Fernsehen oder im Radio war. Und war erstaunt, weil ich nichts davon gewusst habe, dass daran gearbeitet wird, dass das so sein soll (IP7).

Eine Teilnehmerin habe sogar von einer ehemaligen Patientin über die Novelle erfahren und schilderte ihre Frustration ganz explizit:

Ich war extrem verwundert, weil ich als niedergelassene Ärztin von dem überhaupt nichts gewusst habe und war dementsprechend natürlich auch überrascht […] Ich bin vor den Computer, habe gegoogelt, habe mir das angeschaut und habe mir schon gedacht, so in Bezug auf Kommunikation total schlecht gelaufen, weil wenn das so ist, dann hätten wir darüber informiert werden müssen und das ist nicht erfolgt (IP6).

Die fehlende Transparenz während der Entwicklungsphase der Novelle und ein Mangel an Eingebundenheit stellte somit den ersten Kritikpunkt vieler befragten KJPs hinsichtlich der Kommunikationsweise dar. Der zweite wesentliche Kritikpunkt lag in der damit verbundenen fehlenden Vorlaufzeit, also dem sofortigen Inkrafttreten der Novelle. Diesbezüglich wurde von niedergelassenen KJPs betont, dass eine Vorlaufzeit für eine gute Steuerung der eigenen Behandlungskapazitäten notwendig gewesen wäre. Eine Befragte berichtet beispielsweise davon, dass sie am Tag nach der Verkündigung von zehn ehemaligen Patient:innen angerufen worden sei, die dann zum Teil auch vehement einen Termin eingefordert hätten. Die Sorge vor einem übermäßigen Andrang war dabei auch bei jenen KJPs präsent, die später keine bedeutende Zunahme an Anfragen verzeichneten, denn zum Zeitpunkt der Verkündigung der Novelle mussten „*sich [alle] fürchten vor dem Ansturm - der in meinem Fall aber nicht gekommen ist*“ (IP 14). Das Fehlen einer Vorlaufzeit war besonders deswegen so problematisch, weil das kinder- und jugendpsychiatrische Versorgungssystem in Österreich ohnedies an einem akuten Mangel an KJPs leidet [18]. Das zeigt sich auch im niedergelassenen Bereich. So sind laut einer Studie von Koubek et al. [19] aus dem Jahr 2022 etwa zwei Drittel der kassenärztlichen Stellen unbesetzt. Dementsprechend ist es wenig verwunderlich, dass die befragten KJPs durch ihre hohe Auslastung praktisch keine Flexibilität für die Aufnahme zusätzlicher Patient:innen hätten, welche jedoch ihrerseits aufgrund der Novelle diesbezüglich Hoffnung geschöpft hätten und in weiterer Folge enttäuscht wurden. Die unmittelbare Umsetzung der Novelle habe dadurch bei einigen Patient:innen zu einer frustrierenden Absageerfahrung geführt.

### Thema 2: Ende der rechtlichen Grauzone bei hoher fachlicher Akzeptanz: Die Novelle als Legitimierung bewährter Behandlungskontinuität

Trotz der Kritik am Kommunikationsprozess wurde die Novelle auf der fachlich-inhaltlichen Ebene von den Befragten als überwiegend positiv bewertet. Kritisch sei bisher vor allem gewesen, dass der Wechsel von der KJP zur EP mit 18 Jahren zu einem Zeitpunkt geschieht, der im österreichischen Bildungssystem häufig zeitlich mit der entscheidenden Endphase einer Schul- oder Ausbildungsetappe zusammenfällt, deren erfolgreiche Bewältigung bereits ohnedies energie- und zeitmäßig sehr belastend ist.

Ich glaube, dass sie [die Novelle] inhaltlich schon lange notwendig war. Weil diese Grenze mit 18 Jahren eine rein willkürliche war. Das hat in der täglichen Arbeit für den Patienten oft massive Nachteile gehabt. Und wenn jemand, der schon länger in Betreuung ist, im Jänner seines Maturajahres 18 wird und sich dann einen neuen Arzt suchen muss, der diese ganzen Probleme nicht kennt […] dann war das für die Patienten oft ein wirklich schwieriges Problem, in so einer kritischen Phase der eine neue Betreuung zu finden und auch sich wieder ein Vertrauensverhältnis aufzubauen (IP8).

Weiters thematisierten einige Befragte auch den „Kulturschock“, den viele 18-Jährige bei einem Wechsel in die erwachsenenpsychiatrische Versorgung erleben würden. Die Unterschiede in der Behandlungskultur zwischen der KJP und der EP [11] würden viele 18-Jährige teils radikal überfordern. So berichtet eine Befragte von Behandlungsabbrüchen kurz nach dem Übergang in den Erwachsenenversorgung:

Ich habe auch Fälle gehabt, die tatsächlich dann eigentlich nie wirklich so gut angekommen sind im Erwachsenenbereich. Also die dann auch schnell dort wieder abgebrochen haben, obwohl vorher eine beständige Begleitung möglich war und die auch Termine gut einhalten konnten (IP13).

Dass es sich hierbei nicht um Einzelfälle oder ein rein österreichspezifisches Problem handle, zeigt ein stetig wachsendes Maß an Literatur, das die hohen Behandlungsabbruchsquoten und Behandlungslücken am Übergang zu erwachsenenpsychiatrischer Versorgung belegt [9, 20, 21]. Um dieser Problematik proaktiv entgegenzutreten, behandeln einige der Befragten manche ihrer Patient:innen über das 18. Lebensjahr hinaus, obwohl sie sich damit bis zur Novelle in einer rechtlichen Grauzone bewegten:

Für eine junge Erwachsene, die in der Maturavorbereitung ist […] Da verfahre ich generell so, dass ich denen eine Weiterbehandlung bis zum Abschluss der Schulzeit, also sprich mit Erreichen der Matura anbiete, weil dann gibt es eine Neuorientierung, wo sie sich in der Regel dann auch noch mal hinsichtlich eines Studienplatzes neu orientieren und wo es für die Betroffenen meistens einen neuen Lebensentwurf gibt (IP3).

Die Übergangsphase wird von mehreren Teilnehmer:innen als holprig beschrieben. Oft gestalte es sich schwierig, eine passende Weiterbehandlungsmöglichkeit zu finden:

Also bei einer Patientin habe ich drei Anläufe gebraucht, weil die dann die Erwachsenenpsychiater abgelehnt hat. Und dann haben wir wieder schauen müssen, wen gibt es denn noch und wer ist zahlbar? Weil, es gibt ja Leute, die keine Privatärzte nehmen können, sondern halt Kassenvertrag-Ärzte brauchen (IP9).

In manchen Fällen würden jedoch Behandlungen auch seitens der Erwachsenenpsychiater:innen (EPs) abgelehnt werden. Besonders häufig sei dies laut Befragten bei Patient:innen mit Essstörung der Fall, deren Behandlung sich manche EPs aufgrund fehlender Erfahrung mit dem Störungsbild nicht zutrauen würden. Der Forschungsstand zeigt diesbezüglich, dass Wissenslücken und Unsicherheiten bei der Behandlung von Essstörungen auch bei Psychiater:innen in anderen Ländern vorherrschen [22–24].

Generell würden die Befragten versuchen, den Übergang möglichst frühzeitig einzuleiten, so berichtet eine Befragte beispielsweise davon, bereits ein halbes Jahr vor dem 18. Geburtstag den Übergang mit ihren Patient:innen zu thematisieren. Weiters wird berichtet, dass die Novelle KJPs bei schwierigen Übergangsverläufen mehr Autonomie und Zeit bringe, um die bestmögliche Lösung für ihre Patient:innen zu finden. Diese positive Grundhaltung der Befragten gegenüber der Novelle entspricht im Übrigen auch der offiziellen Position der Österreichischen Gesellschaft für Kinder- und Jugendpsychiatrie, Psychosomatik und Psychotherapie (ÖGKJP), wie sie ihr Präsident Univ.-Prof. Dr. Paul Plener auf der Website postuliert:

Wir freuen uns sehr, dass seitens des Gesundheitsministers eine schon länger bestehende Forderung der Österreichischen Fachgesellschaft für Kinder- und Jugendpsychiatrie aufgegriffen wurde und es damit möglich ist, Kontinuität im Behandlungsprozess für unsere Patient:innen zu schaffen [25].

In diesem Statement wird aber auch betont, dass *„dennoch auf die weiterhin begrenzten Ressourcen in der kinder- und jugendpsychiatrischen Versorgung hingewiesen werden [muss]“* [25]. Diese Ressourcenknappheit bildet auch den Kern des nachfolgen Themas, das sich jenen Herausforderungen widmet, die eine Umsetzung des Potenzials der Novelle in der klinischen Praxis komplizieren.

### Thema 3: Wenn die Kleinen Vorrang haben: Ressourcenmangel als Hürde im Rahmen nicht-stationärer Weiterbehandlung

Die niedergelassen KJPs sprachen im Rahmen der Interviews von einer grundsätzlich großen Bereitschaft zur Weiterbetreuung von Volljährigen. Das zeigt sich auch schon an der bereits beschrieben und von manchen auch bereits zuvor gelebten Praxis, 18-Jährige im rechtlichen Graubereich weiterzubehandeln. Angesichts der limitierten Versorgungskapazität [19] sehen sich jedoch viele niedergelassene KJPs gezwungen, ihre Behandlungskapazitäten primär oder ausschließlich für jüngere Patient:innen zu reservieren.

Also ich mache es immer noch so, ich […] schließe meine Behandlungen mit 18 ab, […] ich brauche die Ressourcen und Zeit für die Unter-18-Jährigen. Also es ist ein derartiger Zulauf […] zu uns in die Niederlassung, also ich habe eine Wartezeit, Wartelisten für dringend notwendige Termine für 6‑, 7‑, 8‑Jährige, und da brauche ich einfach die Kapazitäten dafür (IP6).

Ein ähnliches Bild wird von einem Befragten skizziert, der in einem Ambulatorium arbeitet. Auch hier verunmöglicht die bestehende Ressourcenknappheit eine Weiterbehandlung über die Volljährigkeit hinaus:

Wir haben ja bisher schon riesenlange Wartezeiten, Kapazitätsmängel, das heißt, wir bringen ja nicht einmal die Kinder im Kindesalter unter, geschweige denn, wenn wir dann die Kinder noch länger behalten […] also wir wüssten überhaupt nicht, wie wir jetzt die Altersausweitung kapazitätsmäßig unterbringen könnten (IP1).

Die Betrachtung der internationalen Lage verdeutlicht, dass die Ressourcenknappheit im Bereich der KJP kein ausschließlich österreichisches Phänomen darstellt. Studien zeigen ähnliche strukturelle Defizite in anderen europäischen Gesundheitssystemen [26, 27].

### Thema 4: „Es nervt mich“ – Einzelfallprüfungen und informelle Abmachungen statt klarer Regelungen mit den Krankenkassen

Ein weiteres Hindernis stellen die teilweise uneinheitlichen und nicht immer völlig eindeutigen Vereinbarungen mit den örtlichen Krankenkassen dar. Dabei wurden aus den verschiedenen Bundesländern unterschiedliche Erfahrungen bei der Verrechnung der Behandlungskosten nach Erreichen der Volljährigkeit berichtet. In Wien gebe es beispielsweise schon länger eine informelle Abmachung, dass die Österreichische Gebietskrankenkasse (ÖGK) eine Weiterbehandlung bis ca. 21 Jahre vergütet. Befragte aus Niederösterreich gaben an, dass sie sich gegenüber der Krankenkasse immer mittels Schreiben rechtfertigen müssten, wenn man Patient:innen über die Volljährigkeit hinaus weiterbehandeln wolle:

Es nervt mich. Manchmal vergesse ich auch, ich meine ganz ehrlich, man hat ja was anderes zu tun. Dann ein kurzes Schreiben, wobei ich mir denke, ich darf [ja nun weiterbehandeln], warum soll ich da jetzt wieder einen Antrag stellen (IP 11)?

Ein/e Teilnehmer/in aus Vorarlberg berichtet, dass „*die hiesige ÖGK sich zunehmend geweigert hat, die Weiterbehandlung von jungen Erwachsenen bei Jugendpsychiatern zu finanzieren*“ (IP3). Aus Salzburg berichtete eine befragte Person, dass sie aufgrund der trotz Gesetzesnovelle bestehenden Unklarheit hinsichtlich der Finanzierung von selbst sogar einen zuständigen Referenten der Ärztekammer beauftragt habe, bei der ÖGK nachzufragen. Dabei habe sie erfahren, „*dass weiterhin die Entscheidung dem Sachbearbeiter obliegt, ob das dann übernommen wird von der Kasse und es wird als Einzelfall überprüft, wenn jemand über 18 ist*“ (IP14). Aus den Aussagen wird ein klarer Wunsch niedergelassener KJPs nach einer Gesetzesänderung entnommen, die über die bloße Novelle der ÄAO hinausgeht und die Finanzierung der Weiterbehandlung ohne zusätzliche administrative Hürden wie Rechtfertigungsschreiben oder Einzelfallprüfungen durch die Kassen garantiert.

### Thema 5: Volljährige auf der KJP-Station? Eher Minderjährige auf der EP …

Im stationären Bereich wird vor allem der hohe Bettendruck als Kernproblem gesehen, das einer tatsächlichen Weiterbehandlung von gerade volljährig gewordenen Patient:innen auf der KJP im Wege steht. Vor allem in Wien und Niederösterreich arbeitende KJPs kamen auf dieses Thema zu sprechen.

Solange wir so wenige Betten haben, dass wir oft Minderjährige auf die EP legen müssen, solange brauchen wir uns keine Gedanken über eine stationäre Weiterbehandlung von Über-18-Jährigen auf der KJP machen. Ich erinnere mich, dass wir einmal sogar einen 14-Jährigen auf die EP legen mussten, weil es einfach nicht anders gegangen ist (IP12).

Mehrere Befragte beteuern jedoch, dass im Regelfall alles versucht werde, die Anzahl solcher Fälle möglichst gering zu halten, manchmal gebe es jedoch keine andere Option. Ein rezenter systematischer Review zeigt, dass solche Aufnahmen mit erheblichen Herausforderungen verbunden sind: Die Aufenthaltsdauer ist meist deutlich kürzer – was möglicherweise Ausdruck einer arbeitskulturellen Orientierung der EP ist, die im Sinne einer Förderung schneller Re-Integration in die Gesellschaft längere Aufenthalte zu vermeiden versucht – während altersgerechte Angebote häufig fehlen und die Nähe zu akut erkrankten Erwachsenen von Jugendlichen als stark belastend erlebt wird [28].

Häufig bestehe Interesse von Patient:innen-Seite, auch mit 18 Jahren noch auf der KJP behandelt zu werden: „*Also was ich so mitgekriegt habe, würden die alle viel lieber in die KJP kommen“* (IP 10). Möglich sei dies aber nur in absoluten Ausnahmefällen. So wird beispielsweise von einer Klinik berichtet, in der volljährig gewordene Patient:innen mit Essstörungen weiterhin auf der KJP behandelt werden, da für Patient:innen mit diesen Diagnosen kein adäquates Behandlungsangebot auf der EP existiere. In der Zusammenschau wird auch im stationären Bereich ein Ressourcenmangel deutlich, der das Ausschöpfen des Potenzials der Novelle behindert. Im Einklang mit diesen Erzählungen aus der klinischen Alltagspraxis ermittelten Sevecke et al. [29] in einer Studie 2022 eine Bettenmessziffer (BMZ) von 0,04 für stationäre kinder- und jugendpsychiatrische Betten, die den im Österreichischen Strukturplan Gesundheit nach wie vor festgelegten Planungsrichtwert von 0,05–0,09 [29] unterschreitet. Ebenso unterschritt 2022 die tatsächliche BMZ psychosomatischer Betten mit 0,01 [29] den noch heutig gültigen Richtwert von 0,02–0,04 [30]. Diese Zahlen legen nahe, dass die strukturelle Unterversorgung im stationären Bereich eine adäquate Versorgung der Unter-18-Jährigen zu einer großen Herausforderung macht und eine Ausweitung der Behandlungsmöglichkeiten auf ältere Patient:innen nicht realisierbar erscheint.

## Fazit für die Praxis

Zusammenfassend ist festzuhalten, dass die befragten KJPs die Novelle trotz mitunter starker Kritik an der unmittelbaren Inkraftsetzung und der damit fehlenden Vorlaufzeit inhaltlich begrüßen. Der Ressourcenmangel der österreichischen kinder- und jugendpsychiatrischen Versorgungslandschaft lässt es jedoch zum aktuellen Zeitpunkt kaum zu, dass das in der Novelle enthaltene Potenzial verwirklicht werden kann. Dies gilt sowohl für den stationären als auch den nicht-stationären Bereich, da jegliche Form der Weiterbehandlung immer mit den Opportunitätskosten anderer dringend benötigter Behandlungsplätze für jüngere Patient:innen abgewogen werden muss. Die nachhaltige Realisierung des Potenzials der Novelle erfordert somit eine umfassende Investition in personelle und infrastrukturelle Kapazitäten. Ansonsten ist zu erwarten, dass die mit der Novelle ursprünglich angestrebte Verbesserung der psychiatrischen Versorgung im Transitionsalter in der Praxis ausbleibt.

## References

[1] Reed-Fitzke K, Lucier-Greer M. Difficulties in the Transition to Adulthood. In: The Handbook of Systemic Family Therapy. 2020. S. 191–215.

[2] Becht AI, et al. The quest for identity in adolescence: Heterogeneity in daily identity formation and psychosocial adjustment across 5 years. Dev Psychol. 2016;52(12):2010–21. 27893245 10.1037/dev0000245

[3] Harahap A, et al. The Effect of Social Support on Adolescent Mental Health: Literatur Review. J Kedokteran Brawijaya. 2024;p:40–5.

[4] Bacigalupe G, Camara M. The role of social support in adolescents: are you helping me or stressing me out? Int J Adolesc Youth. 2014;22(2):123–36

[5] Fegert JM, et al. Übergang zwischen Jugend- und Erwachsenenalter. Psychotherapeut. 2017;62(1):34–8. 10.1024/1422-4917/a000502 28124949

[6] Höflich A, Schrank B, Aigner M. Herausforderung Transitionspsychiatrie im Rahmen der Erwachsenenpsychiatrie. Psychopraxis Neuropraxis. 2022;25(5):284–8.

[7] Neumann M, et al. Mind the gap – Forderungen für eine verbesserte transitionspsychiatrische Versorgung aus Expert:innenperspektive. Psychopraxis Neuropraxis. 2024;.27:245–50.

[8] Schrank B. Transitionspsychiatrie – ein Definitionsversuch mit Praxisbezug. Psychopraxis Neuropraxis. 2022;25(2):100–2.

[9] Jackson B, Jackson KT, Booth R. “I Fell through the Cracks”: Navigating the Disjointed Transition from Paediatric to Adult Psychiatric Services. Issues Ment Health Nurs. 2022;43(6):507–15. 35025711 10.1080/01612840.2021.2009604

[10] Cleverley K, et al. Identifying core components and indicators of successful transitions from child to adult mental health services: a scoping review. Eur Child Adolesc Psychiatry. 2020;29(2):107–21. 30294756 10.1007/s00787-018-1213-1 PMC7024692

[11] Trabi T, Purtscher-Penz K, Plener P. Versorgung psychisch kranker Jugendlicher im Prozess der Transition vom Jugend- ins Erwachsenenalter. neuropsychiatrie. 2023;37(1):26–32. 36414829 10.1007/s40211-022-00441-0

[12] Pollak E, et al. Transitions- und Adoleszenzpsychiatrie in Österreich: Eine Pilotuntersuchung zur Sicht von Expert(innen). Z Kinder Jugendpsychiatr Psychother. 2017;46(4):325–35. 29183258 10.1024/1422-4917/a000559

[13] Bundesministerium für Soziales, G., Pflege und Konsumentenschutz. Jugendpsychiatrie darf Patient:innen künftig bis zum Alter von 25 behandeln. 2024. https://www.sozialministerium.at/Services/Neuigkeiten-und-Termine/jugendpsychiatrie.html.

[14] Sonderfach Kinder- und Jugendpsychiatrie und Psychotherapeutische Medizin, in BGBl. II Nr. 147/2015, Anlage 14. Republik Österreich. 2024.

[15] Braun V, Clarke V. Reflecting on reflexive thematic analysis. Qual Res Sport Exerc Health. 2019;11(4):589–97.

[16] Braun V, et al. Doing Reflexive Thematic Analysis. In: Bager-Charleson S, McBeath A, Hrsg. Supporting Research in Counselling and Psychotherapy : Qualitative, Quantitative, and Mixed Methods Research. Cham: Springer; 2022. S. 19–38.

[17] Braun V, Clarke V. Supporting best practice in reflexive thematic analysis reporting in Palliative Medicine: A review of published research and introduction to the Reflexive Thematic Analysis Reporting Guidelines (RTARG). Palliat Med. 2024;38(6):608–16. 38469804 10.1177/02692163241234800 PMC11157981

[18] Thun-Hohenstein L, d.A.V.d.. ÖGKJP, Versorgungsituation der Kinder- und Jugendpsychiatrie in Österreich – Stand 2022 (Teil II). neuropsychiatrie. 2023;37(1):1–3. 36847999 10.1007/s40211-023-00461-4

[19] Koubek D, Krönke H, Karwautz A. Die aktuelle Situation der kinder- und jugendpsychiatrischen Versorgung in Österreich im niedergelassenen Bereich. neuropsychiatrie. 2022;36(4):160–4. 36315385 10.1007/s40211-022-00437-w PMC9722836

[20] Loos S, et al. Lost in transition? Perceptions of health care among young people with mental health problems in Germany: a qualitative study. Child Adolesc Psychiatry Ment Health. 2018;12:41. 30093915 10.1186/s13034-018-0249-9 PMC6080358

[21] Reale L, Bonati M. Mental disorders and transition to adult mental health services: A scoping review. Eur Psychiatry. 2015;30(8):932–42. 26647869 10.1016/j.eurpsy.2015.07.011

[22] Jing E, et al. Knowledge and attitudes towards eating disorders: A survey of psychiatrists and psychiatry trainees in New South Wales. Australas Psychiatry. 2024;32(4):375–82. 38705873 10.1177/10398562241249906

[23] Almeida MC, et al. Brazilian psychiatrists’ knowledge of and perceived confidence in eating disorder diagnosis and treatment recommendations. Braz J Psychiatry. 2024;46:e20233516. 38588459 10.47626/1516-4446-2023-3516 PMC11559849

[24] Jones WR, Saeidi S, Morgan JF. Knowledge and attitudes of psychiatrists towards eating disorders. Eur Eat Disord Rev. 2013;21(1):84–8. 23350077 10.1002/erv.2155

[25] ÖGKJP. Endlich Luft nach oben. Österreichische Gesellschaft für Kinder- und Jugendpsychiatrie begrüßt die Gesetzesnovelle zur Behandlung im Transitionsalter. 2024.

[26] Rocha TB, et al. Provision of mental healthcare for children and adolescents: a worldwide view. Curr Opin Psychiatry. 2015;28(4):330–5. 26001925 10.1097/YCO.0000000000000169

[27] Signorini G, et al. Architecture and functioning of child and adolescent mental health services: a 28-country survey in Europe. Lancet Psychiatry. 2017;4(9):715–24. 28596067 10.1016/S2215-0366(17)30127-X

[28] Holland J, et al. The Impact of Child and Adolescent Inpatient Psychiatric Admissions Out-of-Area or to Adult Wards: A Systematic Review. Br J Hosp Med (lond). 2024;85(12):1–20. 39831505 10.12968/hmed.2024.0466

[29] Sevecke K, Wenter A, Böge I. Stationäre Versorgung in der Kinder- und Jugendpsychiatrie – wer hat Platz? neuropsychiatrie. 2022;36(4):179–87. 36348224 10.1007/s40211-022-00443-y PMC9643980

[30] Bundesministerium für Soziales, Gesundheit, Pflege und Konsumentenschutz. Österreichischer Strukturplan Gesundheit 2023. 2023.

